# Interaction of oculomotor and manual behavior: evidence from simulated driving in an approach–avoidance steering task

**DOI:** 10.1186/s41235-019-0170-7

**Published:** 2019-06-13

**Authors:** Norbert Schneider, Lynn Huestegge

**Affiliations:** 1 University of Würzburg, Röntgenring 11, 97070 Würzburg, Germany; 2 Würzburger Institut für Verkehrswissenschaften (WIVW GmbH), Robert-Bosch-Str. 4, 97209 Veitshöchheim, Germany

**Keywords:** Steering, Driving simulation, Gaze control, Visual orientation, Cross-modal action control

## Abstract

**Background:**

While the coordination of oculomotor and manual behavior is essential for driving a car, surprisingly little is known about this interaction, especially in situations requiring a quick steering reaction. In the present study, we analyzed oculomotor gaze and manual steering behavior in approach and avoidance tasks. Three task blocks were implemented within a dynamic simulated driving environment requiring the driver either to steer away from/toward a visual stimulus or to switch between both tasks.

**Results:**

Task blocks requiring task switches were associated with higher manual response times and increased error rates. Manual response times did not significantly differ depending on whether drivers had to steer away from vs toward a stimulus, whereas oculomotor response times and gaze pattern variability were increased when drivers had to steer away from a stimulus compared to steering toward a stimulus.

**Conclusion:**

The increased manual response times and error rates in mixed tasks indicate performance costs associated with cognitive flexibility, while the increased oculomotor response times and gaze pattern variability indicate a parsimonious cross-modal action control strategy (avoiding stimulus fixation prior to steering away from it) for the avoidance scenario. Several discrepancies between these results and typical eye–hand interaction patterns in basic laboratory research suggest that the specific goals and complex perceptual affordances associated with driving a vehicle strongly shape cross-modal control of behavior.

## Significance

Safely driving a car in traffic requires the driver to constantly watch out for relevant information from his/her surroundings and use this information to adapt responses such as operating the accelerator pedal, the brake, or the steering wheel. Occasionally, when a crossing car or pedestrian occurs, a swift steering response of the driver is required to avoid an accident. In these situations, a complex coordination of gaze and manual responses is required. In basic research, the coordination of visual and manual responses and their determinants have been studied extensively. However, little is known about how these findings generalize to applied settings. Specifically, how long it takes a driver to perceive relevant new (visual) information and to select an adequate steering response, and how exactly the corresponding gaze behavior and manual behavior interact, is an open issue. The results of this study indicate that an avoidance task (compared to an approach task) increases oculomotor response times and response pattern variability but not manual steering response times. This finding suggests that highly trained, goal-driven cross-modal motor routines render basic effects of spatial cross-modal response incompatibility practically irrelevant in a driving context. In addition, the study provides estimates of the time needed to initiate directed steering reactions under different task conditions, thereby helping researchers and developers in applied domains to find new ways to increase traffic safety.

## Introduction

Humans usually do not execute actions solely within a single effector system, but constantly coordinate actions across several output domains (e.g., manual, oculomotor, vocal, or other motor control units). This is also true for navigating a car in traffic, where we need to steer the car manually and control the speed with our foot while scanning the environment or controlling the driving trajectory with our eyes. The integration and coordination of behavior across several specific output modalities in response to complex action control demands has been studied under the umbrella term “cross-modal action” (Huestegge & Hazeltine, 2011). However, while cross-modal action has been thoroughly studied in basic research (see later), surprisingly little is known about these phenomena in more applied settings; for example, regarding the interaction of manual steering and gaze behavior while driving. Most existing studies in this domain focused on the interaction of steering and gaze behavior while driving curves (Land & Lee, 1994; Wilson, Stephenson, Chattington, & Marple-Horvat, 2007) and showed that the driver looks in the direction(s) he is going to steer (see also Pfeuffer, Kiesel, & Huestegge, 2016, for basic mechanisms underlying anticipatory oculomotor control), whereas other studies analyzed the influence of cross-modal action between verbal tasks (i.e., talking on a phone) and (spatial) visual attention while driving (e.g., Atchley, Dressel, Jones, Burson, & Marshall, 2011). However, these results are not informative regarding the issue of gaze–steering interaction when drivers respond to suddenly appearing objects, which require a swift response from the driver. Specifically, to date little is known about how long it takes the driver to show a steering reaction to sudden hazardous events and how gaze behavior (spatially and temporally) interacts with steering behavior. The present study intends to fill this gap by providing a first approach to this topic. In the following, we will summarize previous findings about driver reactions to suddenly appearing objects, and about basic mechanisms of cross-modal action control with a focus on the interaction of gaze behavior and manual responses.

### Driver reactions to suddenly appearing objects

Previous studies suggest that drivers often tend to prefer braking over steering to avoid an accident, sometimes even in situations where steering might be the better collision avoidance strategy (Adams, 1994; Adams, Flannagan, & Sivak, 1995; Dozza, 2013; Malaterre, Ferrandez, Fleury, & Lechner, 1988; Malaterre & Lechner, 1990; McGehee et al., 1999; Wiacek & Najm, 1999). However, in those cases where drivers try to avoid an obstacle by steering, they tend to swerve in the moving direction of the obstacle (Scanlon, Kusano, & Gabler, 2015; Weber & Färber, 2015; Wiacek & Najm, 1999). According to Malaterre and Lechner (1990) and Weber and Färber (2015), the time to collision (TTC) strongly influences the likelihood of an evasive steering maneuver. Specifically, their data suggest a U-shaped correlation, in that the likelihood for an evasive maneuver increases if the TTC is smaller than 1.8 s or greater than 2.2 s.

So far, most studies reporting steering reaction time (RT) have been conducted in laboratory settings (Müsseler, Aschersleben, Arning, & Proctor, 2009; Proctor, Wang, & Pick, 2004; Wang, Proctor, & Pick, 2003) and report data on manual steering responses to spatially presented visual or auditory stimuli. More specifically, they have focused on the influence of spatial compatibility between the position of the imperative stimulus and the required steering reaction. Spatial compatibility is a special form of stimulus–response (S–R) compatibility, which is thought to be based on the spatial association between a stimulus and the required response (Proctor & Vu, 2006). In general, high (spatial) S–R compatibility is assumed to yield a shorter RT than low (spatial) S–R compatibility. This prediction does not only hold for task-relevant stimulus features (e.g., pressing a right key in response to a stimulus on the right), but also for task-irrelevant stimulus features; for example, when a stimulus requiring a right key press is displayed on the right (vs left) side of a display although the stimulus presentation location is not task relevant (“Simon effect”; Simon, 1969; Simon & Rudell, 1967).

In the context of traffic psychology, both Müsseler et al. (2009) and Wang et al. (2003) found that spatially compatible stimuli (i.e., stimuli that are spatially compatible with the required steering response) lead to faster steering RTs compared to spatially incompatible stimuli, with an advantage of approximately 60 ms (Wang et al., 2003) or 15 ms (Müsseler et al., 2009), while steering RTs ranged from 425 to 625 ms. Interestingly, however, Müsseler et al. (2009) also showed that spatial compatibility effects can be reversed in specific driving situations: participants responded faster (about 21 ms) when they steered away from a pedestrian (spatially incompatible response) compared to steering toward a pedestrian (spatially compatible response). They assumed that such a reversed compatibility effect is the consequence of stimulus valence, which might be associated with a corresponding response (e.g., avoidance of stimuli with “negative”, hazard-related valence and approach toward stimuli with "positive" valence). This indicates that mere spatial S–R compatibility might only exert a negligible influence in driving situations, where the driver has specific goals and intentions which are associated with the driving task and depend on experience and training (e.g., avoid collisions while driving). Consequently, the avoidance response—although spatially incompatible with the stimulus—might eventually be carried out faster because of its congruency with the drivers’ current goals in the context of a highly trained driving task (i.e., goal congruency might override spatial compatibility).

The reversed compatibility effect is also interesting because in real driving situations the avoidance reaction might be more complex than the approach reaction. Specifically, to select the appropriate reaction, drivers must perceive and identify the stimulus first, a process that—despite the possibility that humans can in principle covertly shift attention without any eye movements—should usually be accompanied with corresponding oculomotor behavior (Findlay & Gilchrist, 2003). In the case of a stimulus requiring the driver to steer toward it (approach), drivers can focus on this stimulus during the entire process of preparing and executing the steering response and might not have to divert their visual attention to plan and control the required vehicle trajectory. In this case, the required direction of the attentional (i.e., typically oculomotor) and manual response is the same (cross-modal congruency). In the case of a stimulus requiring the driver to steer away (avoid), however, drivers need to attend to the stimulus as well (in order to respond to it), but also need to manually initiate the required evasive vehicle trajectory in the opposite direction. In this case, the spatial direction of (initial) visual processing and manual responding differs (cross-modal incongruency). How oculomotor and manual control interacts in such situations is an as yet unresolved issue.

### Cross-modal action control: basic mechanisms of the interaction of gaze and manual actions

In basic cognitive research, studies focusing on cross-modal action control have shown that gaze behavior can interfere with concurrent manual actions (Hodgson, Müller, & O’Leary, 1999; Huestegge & Adam, 2011; Huestegge & Koch, 2009), a finding that can be considered a special case of performance costs associated with multitasking (Pashler, 1994). For example, Hodgson et al. (1999) and Huestegge and Koch (2009) showed that both manual and gaze RTs are delayed under simultaneous gaze and manual response demands (see also Huestegge, 2011; Huestegge, Pieczykolan, & Koch, 2014; Tibber, Grant, & Morgan, 2009). These dual-response costs (i.e., additional time to initiate a response in the context of another response vs alone) typically increase when one or both responses are incompatible with the stimulus or incompatible among each other (see Huestegge, 2011). Such performance costs for incompatible responses across effector systems do not only occur for explicitly instructed saccades in the context of manual responses, but also for incidental (not explicitly instructed) saccades (Huestegge & Adam, 2011). Across all of these basic research studies, manual responses were associated with greater dual-response costs and incompatibility effects than the gaze responses. Additionally, the oculomotor response was typically initiated earlier than the manual response under both compatible and incompatible response requirements. Further studies have replicated these findings and suggested a general prioritization of oculomotor responses over manual responses (oculomotor dominance; Huestegge & Koch, 2013; Pieczykolan & Huestegge, 2014). Hodgson et al. (1999) assumed that manual and visual responses might share a common attentional representation of space, which could be an explanation of why especially spatially incompatible motor programs of these different effector systems interfere so strongly with each other. In sum, these studies indicate a possible response delay, especially for manual responses, when incompatible oculomotor and manual responses have to be executed at close temporal proximity in a driving situation, and a general tendency to execute saccades prior to manual responses. However, these predictions have not yet been tested, and it is possible that the general goal to optimize vehicle control may yield quite different control strategies in complex driving situations than under more basic task demands in reduced laboratory settings.

### The present study

With this study, we wanted to analyze the interaction of gaze and manual steering responses while driving as an applied example of cross-modal action control. More specifically, we tried to narrow down the gap between laboratory research and applied research by developing a more realistic yet still standardized experimental setting in a dynamic driving simulator, which incorporates the experimental setup in a driving task. A general question is whether typical result patterns found in reduced, highly controlled basic research settings reflect fundamental cognitive mechanisms that thereby also generalize to more complex real-life tasks (e.g., navigating through traffic). In contrast, it is possible that many effects found in basic research setups are absent (or at least strongly modulated) in more complex, realistic environments due to a strong adaptivity and flexibility of cognitive sets based on changing situations, task demands, and goals.

As already mentioned, Huestegge and Adam (2011) showed that that even mere incidental (as opposed to explicitly instructed) saccades during the preparation of concurrent manual responses significantly affected manual RTs in terms of *spatial congruency effects*: if the direction of the saccade was compatible with the position of the required manual response (cross-modal action congruency), the manual RT was faster compared to trials in which the saccade was incompatible (cross-modal action incongruency). Assuming that such basic laboratory findings generalize to more complex settings and goals, one would expect that such cross-modal action incongruency should also negatively affect steering RT in driving situations, especially when drivers have to steer away from (avoid) a suddenly appearing object (assuming that the driver should usually gaze at the object in order to process it). Conversely, in situations where the driver must steer toward (approach) an object, faster steering RTs would be expected due to cross-modal action congruency (in addition to spatial S–R congruency).

However, in a driving context, such an effect might also be counteracted by the particular goals of a driver. The results of Müsseler et al.’s (2009) study indicated that it is also reasonable to expect no such difference in steering RTs because in both (approach and avoidance) conditions, spatial congruency emerges between the steering movement and the current goal in terms of the intended driving direction. This more high-level, conceptual spatial congruency may override any low-level cross-modal action (and S–R) congruency effects (and probably also affect gaze behavior in general, see following hypothesis).

Second, based on the many laboratory studies already referred to, one might expect that after stimulus onset an oculomotor response should usually be initiated *prior to* the manual response due to the well-known general latency differences between these effector systems that were observed regardless of any particular S–R or R–R congruency conditions (oculomotor dominance in cross-modal dual-response tasks; Huestegge & Koch, 2013). In a driving context, it is furthermore reasonable to assume that the stimulus must be perceived (typically by looking at it) before an appropriate manual steering response will be initiated.

However, it is also possible that covert attention (i.e., without observable eye movements) is used for stimulus processing, especially in avoidance situations where the manual steering trajectory should be planned away from the stimulus, and where oculomotor control may predominantly be devoted to planning and monitoring an optimal vehicle trajectory. Thus, it is entirely possible that avoidance situations are associated with fewer saccades toward the stimulus (i.e., saccades dedicated to stimulus decoding), but more (slightly delayed) saccades in the intended steering direction (for planning/monitoring the steering responses). Since these saccades would serve a different goal, they might well be executed *after* the initial manual steering response. Thus, again, we consider it possible that a robust finding from basic research (here: with respect to *cross-modal response sequence effects*) might not generalize (or at least be strongly modulated) in a complex driving situation due to different underlying goals of the subject.

Third, we wanted to explore whether the combination of both response demands (i.e., mixing of approach and avoidance demands within one experimental block of trials) has an influence on the response pattern and the coordination of oculomotor and manual responses. In real-life situations, drivers might often have to choose between several response options (e.g., steer toward or away from an upcoming target) and to select the correct option (in relation to current task goals) within a short timeframe. Therefore, it is important to implement an environment which requires the driver to dynamically adapt his/her response strategies in accordance with the specific stimulation conditions. Based on findings from basic cognitive research (e.g., Kiesel et al., 2010), we expected typical performance effects associated with experimental blocks involving task switches compared to blocks involving a constant task; that is, an increase in RTs and error rates for blocks requiring both approach and avoidance tasks. Again, we reasoned that even though detrimental *effects of task switching* are well replicated across innumerable basic laboratory experiments, it is important to explicitly test to what extent corresponding effects can also be relevant in more complex real-life task demands such as driving a vehicle.

## Materials and methods

### Participants

Twenty participants (10 female) took part in the study and were paid for their participation. Their mean age was 34.8 years (minimum = 22; maximum = 64 years). The mean self-reported annual driving experience was 9864 km (SD = 9540 km) ranging from 700 to 35,000 km/year. All participants were well trained in the dynamic driving simulator.

### Apparatus

**Fig. 1:**
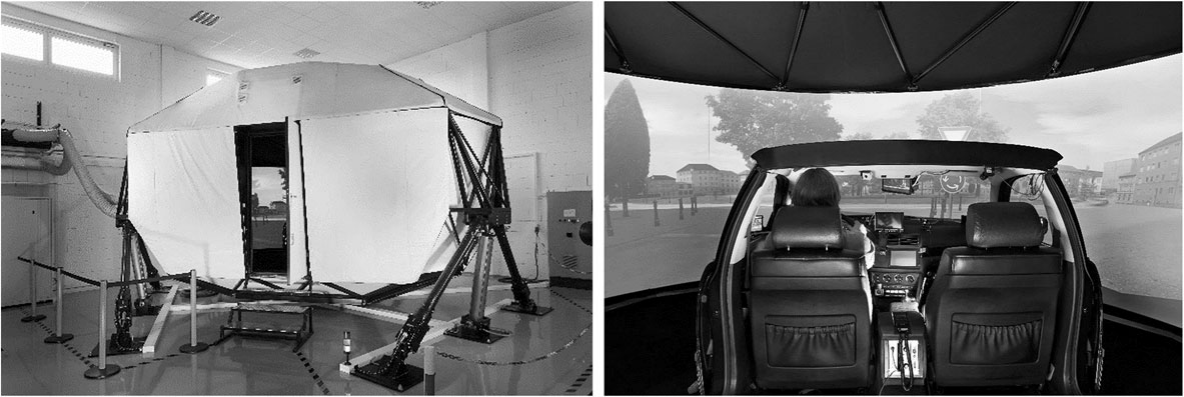
Setup of the driving simulator with the motion platform and the dome (left) and the mock-up inside the dome (right)

The study took place in the dynamic driving simulator of WIVW GmbH (Würzburg Institute for Traffic Sciences GmbH, Würzburg, Germany). The simulator consisted of a dome that was mounted on an FCS Moog motion system with six degrees of freedom. The mock-up was made up of a real BMW 520i that was cut off behind the B-pillar (see Fig. 1).

The simulator had a 180° horizontal and 47° vertical field of vision with three image channels. The image was presented on a spherical projection screen with a diameter of 6 m. There were three LCD displays representing the rear-view mirror and the left and right outside mirrors. Auditory output included eight sound channels including a subwoofer and a shaker. The gaze behavior was recorded with a remote eye tracking system (SmartEye, Sweden). In total, four fixed-base cameras mounted on the dash board and the middle console were used with a sampling frequency of 60 Hz.

The driving simulation software SILAB 5.0 manufactured by WIVW GmbH was used. During simulation, a graphical user interface allowed the observation and logging of all data. The recording had a temporal resolution of 100 Hz. An experimenter observed all driver views on separate display screens and communicated with the participants via an intercom.

### Test tracks and stimuli

**Fig. 2:**
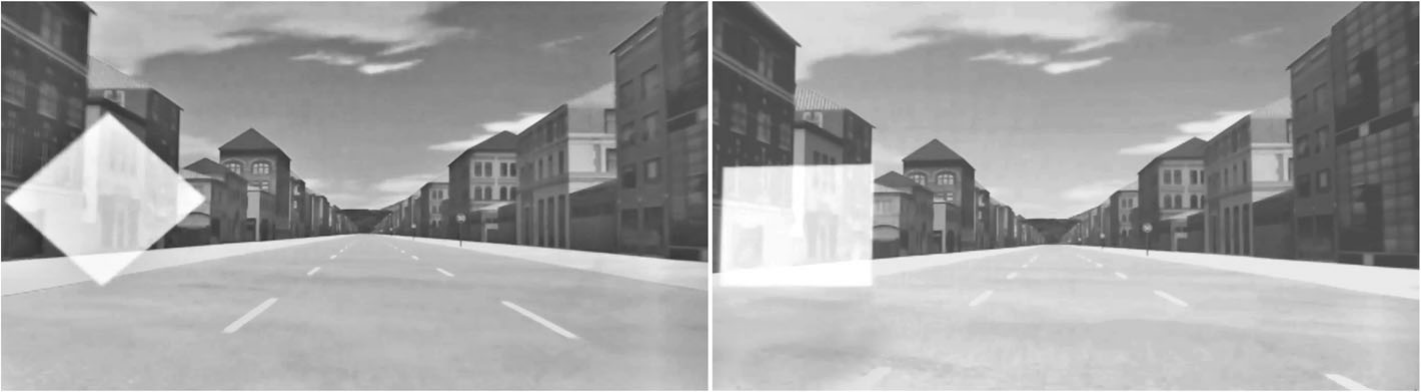
Example design of the test track and the stimuli. Both stimuli, diamond (left) and square (right), could be presented either on the right-hand or left-hand lane

In total, four different test tracks were used for the study: a training track (to familiarize participants with the task, setup, and stimuli); two test tracks with only one stimulus type (i.e., either only approach stimuli or only avoid stimuli, in the following referred to as constant tasks); and one test track with both stimulus types (mixed task). Each test track consisted of a straight road with three lanes (lane width of 3.5 m each). The imperative stimuli were either a yellow semitransparent square or diamond (i.e., a square rotated by 45°). They were presented at a time to collision (TTC) of 1.6 s on either the right-hand or left-hand lane (see Fig. 2) and moved slightly toward the middle lane to increase response affordance of the situation. Although this stimulus movement introduced an additional spatial dimension (movement direction) which is opposed to the stimulus location, we reasoned that this design feature provides a more realistic situation. In addition, any processing of the stimulus necessarily requires an attention shift toward the object to adapt the required steering movement.

Within constant-task test tracks, either 10 squares or 10 rotated squares were presented. In these blocks, the respective stimulus was presented five times on the left-hand lane and five times on the right-hand lane in a predefined random order. Within the mixed-task test track, each stimulus type was presented three times on the left-hand lane and three times on the right-hand lane in a predefined random order. Note that the restricted number of trials (compared with typical trial counts in basic research experiments) was necessary to limit the participants’ time in the simulator to avoid simulator sickness.

### Procedure

**Fig. 3:**
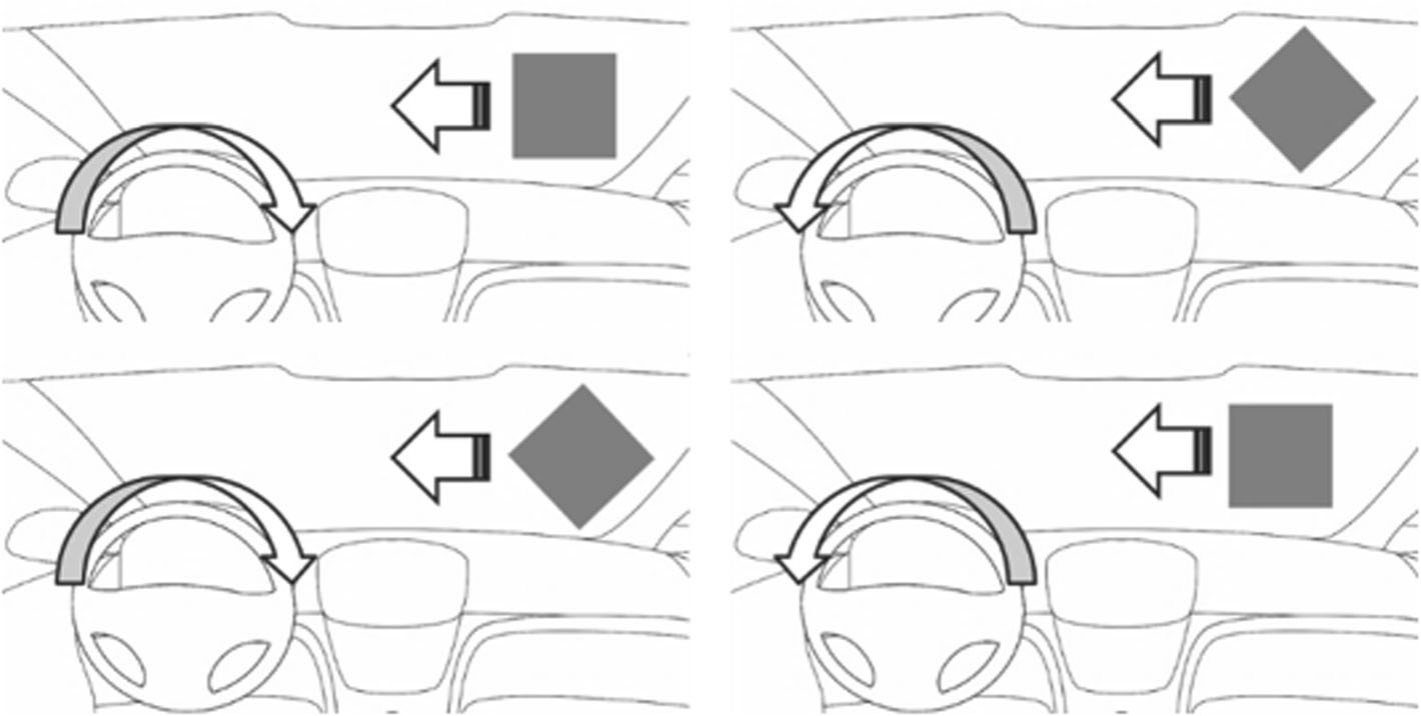
Required steering reactions for the instructions. Required steering reactions for the instruction “approach square” and “avoid diamond” (top) and for the instruction “approach diamond” and “avoid square” (bottom). Note that in the actual experiment, stimuli also occurred on the left

The sample of participants was divided into two groups. Half of the participants received a written instruction which told them to steer toward the square when it appeared (approach square) and to steer away from the diamond (avoid diamond) as quickly and precisely as possible. The other half received the opposite instruction (steer away from the square and toward the diamond, see Fig. 3).

To ensure correct understanding of the instruction and to train the required reactions, participants first completed the training track. Participants then encountered the two constant track conditions. Prior to each of these test tracks, the experimenter told the participant which stimuli would appear and repeated the instruction when required. To control for sequence effects, the order of the two constant test tracks was counterbalanced. After finishing both constant conditions, participants completed the mixed condition (i.e., the mixed condition was always implemented after the constant conditions to avoid carry-over effects that were considered likely to occur with reversed order; see “Limitations” section in Discussion for more details). Prior to the mixed condition, participants were told that both stimuli could appear in this track and were reminded again to react as quick and precisely as possible. In total, the experiment took approximately 30 min to complete.

### Design

The independent within-subject variables were the instructed steering task (approach vs avoid) and block type (constant vs mixed tasks within a block). The dependent variables were steering and gaze RTs (for correct trials only) as well as errors. The steering RT was computed by identifying the onset of the first continuous steering wheel movement after the presentation of the stimulus, based on the steering wheel angle velocity. The gaze RT was computed by identifying the first shift of the gaze heading after the presentation of the stimulus. Complete steering errors were defined as trials in which no instruction-congruent steering movement was observed at all. However, some trials which eventually resulted in a correct steering movement were additionally characterized by a brief initial steering movement in the wrong direction prior to the execution of the final, correct steering movement. Given that the occurrence of such trials covaried with experimental conditions (see Results) and likely represents action selection difficulties, we considered this behavior psychologically meaningful and incorporated these cases into our analyses of steering behavior. Additionally, since these trials involved two manual movement onsets, we discarded these trials in follow-up analyses of the temporal coordination of both responses to achieve a nonambiguous estimate of temporal inter-response intervals that is comparable across conditions.

Additionally, the gaze strategy and coordination of gaze and steering reactions was analyzed. To this end, the gaze behavior was classified as goal congruent (required steering direction and observed gaze direction were the same) or as goal incongruent (required steering direction and observed gaze direction differed). Finally, we analyzed whether the gaze reaction preceded the steering reaction (“gaze first steering second”) or vice versa (“steering first gaze second”). Cases without temporal difference between the steering and gaze RTs were classified as “parallel”.

## Results

### Steering errors

Complete (uncorrected) steering errors rarely occurred (constant blocks, 0% in both “approach” and “avoid” conditions; mixed blocks, 3.6% for “approach” and 7.4% for “avoid”), suggesting that participants were able to follow the instructions. As mentioned earlier, some trials involved an initial steering movement in the wrong direction that was corrected later. Specifically, most drivers adapted their steering reaction within the first second after stimulus presentation with a mean time of 228 ms (SD = 87 ms) between the initially erroneous and the subsequent corrected steering response. Nevertheless, we reasoned that such initial steering errors (along with complete errors) may also indicate action selection difficulties, and therefore included these trials in the final analysis of (initial) steering errors. The resulting (initial) steering error rates amounted to 1.5% (equivalent to *N* = 3 steering movements across all participants and trials) for the instructed steering movement “approach” and 5% (*N* = 10) for the instructed steering movement “avoid” in the constant-task blocks. In the mixed-task blocks, (initial) steering error rates were 46.7% (*N* = 56) for the instructed steering movement “approach” and 45% (*N* = 54) for the instructed steering movement “avoid”. A repeated-measures analysis of variance (ANOVA) of these (initial) steering errors (regarding error rates) revealed a significant effect of block type (*F*(1,19) = 144.312, *p* < 0.001, *η*^2^ = 0.884) but not of steering task (*F*(1,19) = 0.096, *p* = 0.760, *η*^2^ = 0.005), and there was no significant interaction (*F*(1,19) = 1.068, *p* = 0.314, *η*^2^ = 0.053). Note that for a maximally unambiguous estimation of steering RTs, all trials with erroneous initial steering movements were excluded from most of the further analyses of steering RT and gaze RT.

### Steering RT

**Fig. 4:**
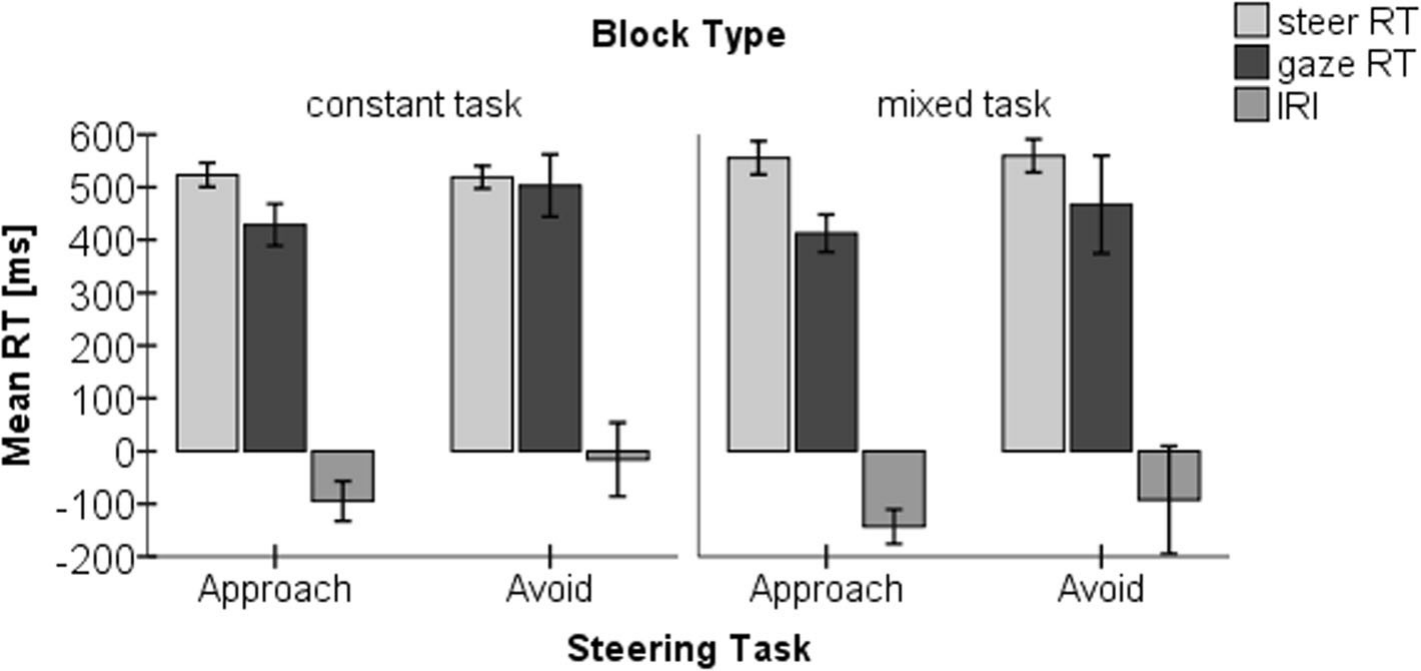
Overview of all reaction time data. Mean steering RT, gaze RT, and IRIs (s) dependent on task (avoid vs approach) and block type (constant task vs mixed task). Error bars represent 95% confidence intervals. IRI inter-response time interval, RT reaction time

This analysis is based on trials with correct (initial) steering direction, irrespective of the direction of the initial oculomotor response. Figure 4 depicts an overview of all RT data. A repeated-measures ANOVA revealed a significant effect of block type (*F*(1,19) = 16.106, *p* = 0.001, *η*^2^ = 0.459) but not of steering task (*F*(1,19) = 0.488, *p* = 0.493, *η*^2^ = 0.025), and there was no significant interaction (*F*(1,19) = 0.399, *p* = 0.535, *η*^2^ = 0.021). The mean steering RT was increased by about 30–40 ms in mixed (*M*_approach_ = 556 ms, *M*_avoid_ = 560 ms) vs constant (*M*_approach_ = 523 ms, *M*_avoid_ = 519 ms) blocks. To ensure that the exclusion of nearly 50% of the data in the mixed block in this analysis did not influence the results, the analysis was repeated with all of the steering RT data (i.e., also the corrected steering trials) included (using the timepoint of the initial erroneous steering movement as the basis for determining the RT). Again, the ANOVA showed a significant main effect of block type (*F*(1,19) = 15.537, *p* = 0.001, *n*^*2*^ = 0.450) but no significant main effect of steering task (*F*(1,19) = 0.098, *p* = 0.758, *n*^*2*^ = 0.005), and there was again no significant interaction (*F*(1,19) = 4.283, *p* = 0.052, *n*^*2*^ = 0.184). In addition, an explorative post-hoc analysis was conducted for the mixed block to test whether the steering RT differed for initially correct vs initially erroneous steering movements. The ANOVA did not reveal significant effects of initial steering movement (correct vs initially erroneous) on the steering RT (*F*(1,19) = 2.513, *p* = 0.129, *η*^*2*^ = 0.117), although it should be noted that this result should be interpreted with care given the low number of trials involved.

### Gaze RT

This analysis is based on the time between stimulus presentation and the first shift of the gaze heading to the left or to the right*.* The ANOVA revealed a significant effect of steering task (*F*(1,18) = 5.814, *p* = 0.027, *η*^2^ = 0.244) but no significant effect of block type (*F*(1,18) = 0.968, *p* = 0.338, *η*^2^ = 0.051). Again, there was no significant interaction (*F*(1,18) = 0.188, *p* = 0.523, *η*^2^ = 0.010). The mean gaze RT was increased by about 60–70 ms for the avoid (*M*_constant_ = 503 ms, *M*_mixed_ = 467 ms) vs approach (*M*_constant_ = 429 ms, *M*_mixed_ = 412 ms) tasks.

To further analyze the influence of the experimental factors on steering and gaze coordination, a repeated-measures ANOVA was conducted for inter-response time intervals (IRIs), defined as the temporal interval between steering and gaze reaction. This analysis indicated significant effects of task (*F*(1,18) = 4.623, *p* = 0.045, *η*^2^ = 0.204) and of block type (*F*(1,18) = 5.286, *p* = 0.034, *η*^2^ = 0.227), whereas there was no significant interaction (*F*(1,18) = 0.424, *p* = 0.523, *η*^2^ = 0.010). Specifically, IRIs were greater in mixed-task vs constant-task blocks (− 117 ms vs − 56 ms), and greater in “approach” vs “avoid” conditions (− 119 ms vs − 55 ms) (see Fig. 4).

### Initial gaze behavior

**Fig. 5:**
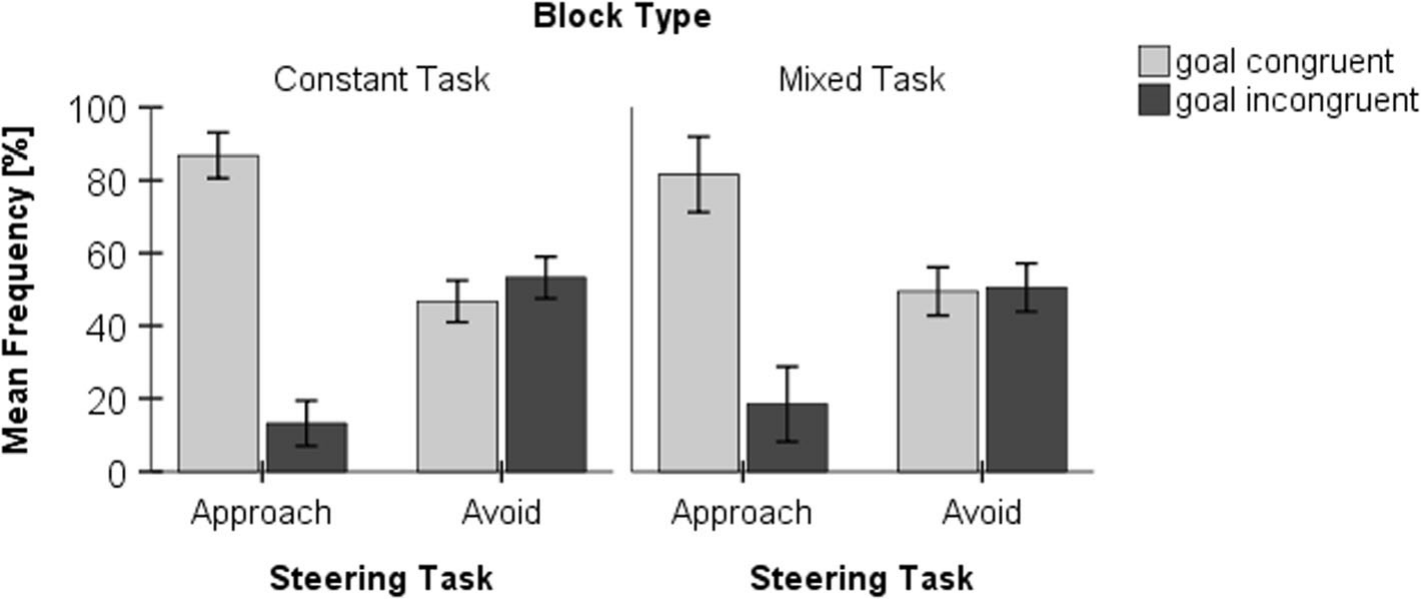
Gaze behavior as a function of steering task and block type. Mean frequency of trials with goal-congruent and goal-incongruent gaze behavior as a function of steering task (approach vs avoid) and block type (constant vs mixed task). Error bars represent 95% confidence intervals

In approach conditions, goal-congruent gaze behavior was predominantly used in both block types by the drivers (constant task, 87%; mixed task, 82%), suggesting that the direction of the initial gaze was the same as the direction of the required steering reaction (see Fig. 5). In avoid conditions, however, we observed goal-congruent (constant task, 47%; mixed task, 49%) and goal-incongruent (thus stimulus-congruent) gaze behavior in about half of the cases (see Fig. 5). A repeated-measures ANOVA regarding the relative frequency of goal-congruent gaze behavior revealed a significant effect of steering task (*F*(1,18) = 76.420, *p* < 0.001, *η*^2^ = 0.809) but not of block type (*F*(1,18) = 0.166, *p* = 0.688, *η*^2^ = 0.009), and there was no significant interaction (*F*(1,18) = 1.971, *p* = 0.177, *η*^2^ = 0.099). This indicates a strong influence of the instructed steering task on gaze behavior. Note that there were too few trials to conduct a statistically interpretable analysis of RTs as a function of response congruency.

### Temporal coordination of steering and gaze behavior

**Fig. 6:**
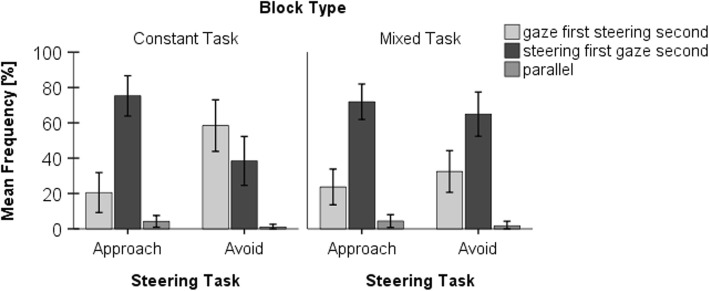
Temporal sequence of steering and gaze behavior dependent on task and block type. Mean frequency of trials with the observed temporal sequence of steering and gaze behavior dependent on task (approach vs avoid) and block type (constant task vs mixed task). Parallel indicates that gaze and steering RT are initiated simultaneously, “gaze first steering second” indicates that the gaze response preceded the steering response (and vice versa for “steering first gaze second”). Error bars represent 95% confidence intervals

An analysis of the temporal order of the observed steering and gaze behavior showed substantial effects of steering task specifically in constant-task blocks (see Fig. 6). In “approach” conditions, the drivers predominantly showed a gaze reaction prior to steering initiation (parallel response initiation, 4%; gaze first, steering second, 75%; steering first, gaze second, 21%). In contrast, in “avoid” conditions a steering reaction was observed prior to a gaze shift in almost two-thirds of the trials (parallel, 1%; gaze first, 39%; steering first, 60%). In mixed-task blocks this difference was no longer present. Under both task conditions, “approach” (parallel, 4%; gaze first, 72%; steering first, 24%) and “avoid” (parallel, 2%; gaze first, 65%; steering first, 33%), the drivers predominantly showed a gaze reaction prior to a steering reaction (see Fig. 6). A repeated-measures ANOVA regarding the relative frequency of the “steering first, gaze second” sequence revealed a significant effect of steering task (*F*(1,18) = 21.218, *p* < 0.001, *η*^2^ = 0.541) and of block type (*F*(1,18) = 7.604, *p* = 0.013, *η*^2^ = 0.297), and a significant interaction (*F*(1,18) = 12.014, *p* = 0.003, *η*^2^ = 0.400).

## Discussion

The present study focused on the interaction of oculomotor gaze and manual steering behavior in approach and avoidance tasks while driving a vehicle. First, based on corresponding effects in basic cognitive research, we aimed at testing whether approach (vs avoid) conditions yield faster manual steering responses, either due to S–R congruency between stimulus position and response direction (e.g., Wang et al., 2003) or due to R–R congruency between (incidental) oculomotor and (instructed) manual responses (Huestegge & Adam, 2011). Specifically, we reasoned that approach tasks should more likely involve R–R congruency, whereas avoid tasks should increase the potential for R–R incongruency. However, the results indicated no significant differences in manual steering RT, suggesting that S–R and R–R congruency had no clearly measurable effect on manual steering response speed. Thus, we could neither replicate the S–R compatibility effects on steering RT reported by Wang et al. (2003) nor any reversed S–R compatibility effects (Müsseler et al. (2009). Probably, the more advanced simulation software in the present study (compared with the previous studies) substantially increased immersion in the driving task, so that the specific goals related to the task at hand were more salient and counteracted basic compatibility effects typically found in more reduced laboratory settings. Interestingly, however, we observed significant effects on the gaze RT, which was prolonged in avoid (vs approach) conditions. This effect, which also shows that our design was principally suited to pick up the effects of task instructions on RT, might be based on qualitative differences in the coordination of oculomotor and manual actions depending on the required response type. This coordination was addressed more elaborately with our second research question.

Based on the findings of several basic laboratory studies (e.g., Huestegge & Adam, 2011; Huestegge & Koch, 2013), we expected that the oculomotor response should be initiated prior to the manual response. Additionally, we assumed that covert attentional processes (i.e., without overt eye movements) might play a role in avoidance tasks (especially in constant-task blocks). Specifically, drivers might generally tend to process the stimulus first and plan the driving trajectory afterward. In approach situations, both subgoals can be served with an early saccade in the stimulus direction, whereas in avoidance situations the first goal might rely on covert attention shifts (for stimulus decoding) while the second subgoal is guided by a corresponding saccade occurring later in time. Thus, avoiding saccades to stimuli might represent a parsimonious action control strategy in avoid conditions to prevent unnecessary oculomotor action. In line with this reasoning, the change of gaze direction was—in the majority of the cases—observed prior to the onset of the steering reaction in approach tasks, while in avoidance tasks changes of gaze direction were registered after the steering reaction in about 60% of the cases (also see corresponding changes in IRI data). Additionally, the delayed gaze response in avoid situations could also be explained by assuming that in these conditions drivers must choose between looking at the stimulus or in the direction they are going to steering (choice reaction task). In contrast, approach conditions do not require such a choice and therefore resemble simple response tasks, which (due to the lack of selecting between several response alternatives) are known to be executed faster (e.g., Sanders, 1980). Finally, we could also observe different gaze strategies. In approach tasks, goal-congruent gaze behavior was dominant, whereas in avoid tasks, both goal-congruent and goal-incongruent gaze behavior was prevalent, which indicates that the avoid task requires a more complex coordination of oculomotor and manual responses than the approach task.^1^

With our third research question we explored whether the combination of both task demands (approach and avoid) affected oculomotor and manual response control. In line with basic research literature suggesting that switching between task demands leads to decreased performance (e.g., Kiesel et al., 2010), we indeed found a significant increase of manual steering RT in mixed (vs constant) blocks, irrespective of task (approach or avoid). Additionally, error rates were also substantially increased in mixed (vs constant) blocks. Several mechanisms are usually discussed for such performance decrements in task switching (Monsell, 2003). For example, it was assumed that any task switch requires a reconfiguration of the involved cognitive control settings, also known as a task set. This reconfiguration is assumed to take time, thereby increasing RTs. Additionally, it is possible that working memory demands related to keeping two (conflicting) task sets active at the same time in working memory is resource-consuming and thus prolongs RTs. Finally, the observed difference in steering RT could also be associated with different task requirements. To select the correct response in mixed-task conditions, participants had to process stimulus shape, whereas in constant-task conditions, they could principally initiate their response solely based on stimulus location. This additional identification of stimulus features might also require more time and could therefore be another cause for the increased steering RT in mixed-task conditions. To selectively test these different potential mechanisms, further research is needed. Especially, switch costs *within* mixed-task blocks could be analyzed with an adapted study design involving more trials in order to obtain reliable measures of switch costs (in terms of sequential effects). In addition, it could be tested whether intensive training could eliminate the substantial increase of error rates and the increased RT in mixed blocks.

Finally, further analyses of the coordination pattern between oculomotor and manual responses revealed an influence of task condition (constant vs mixed) under avoid instructions. In constant-task blocks, drivers usually showed a steering reaction prior to their gaze shift, whereas this pattern was reversed in mixed-task blocks. This supports our assumption that the stimulus must be inspected more closely before initiating a (correct) manual response, especially in mixed-task blocks, where participants were unable to anticipate stimulus identity (and thus the task to be executed).

### Implications for applied research

Based on the present findings we can estimate how long it takes drivers to process relevant information before executing a specific steering response. This could be relevant for the development of steering and evasion assistants, which focus on events in which the driver has only limited time to react and in which a collision can no longer be avoided by braking alone. Several studies indicate that collisions often cannot be avoided by steering/evasion assistants due to counter-steering reactions of the driver (Bräuchle, Flehming, Rosenstiel, & Kropf, 2013; Fricke, Griesche, Schieben, Hesse, & Baumann, 2015; Hesse et al., 2013; Schieben, Griesche, Hesse, Fricke, & Baumann, 2014). However, these studies provide no further explanation of why drivers exhibit counter-steering reactions. We suppose that drivers reduce the effectiveness of these systems because they did not have enough time to analyze the situation and to decide how to respond (e.g., steering to the left/right, or braking). Consequently, automatic steering interventions might be suppressed by the drivers until they have had enough time to decide how to respond, which according to our findings could take up to 600 ms.

Furthermore, our study might help to explain why drivers in general do often not consider evasive steering reactions to avoid collisions (see Adams, 1994), and preferably initiate braking responses instead. Specifically, steering reactions have more degrees of freedom than braking reactions and additionally require a complex coordination of oculomotor and manual responses. Therefore, drivers might resort to the simpler, highly trained braking reaction (see Malaterre et al., 1988).

### Limitations

The present study has several potential limitations. First, the specific task utilized here (fast steering movements) and the dynamic driving simulator setup increases the risk of developing kinetosis, so that we had to deal with strong constraints regarding the number of trials per experimental condition (which also prevented us from analyzing trial-by-trial switch costs). Nevertheless, we were still able to observe several large effects of experimental conditions on performance, while other effects were clearly far from significant (e.g., regarding steering RTs). This suggests that our design was, despite the overall low number of trials, reasonably sensitive to detect important effects on gaze and steering behavior.

Second, we resorted to a design involving a fixed sequence regarding the constant and mixed blocks. While such a fixed block sequence is also typical for basic dual-task and task switching research, where training of component (single) task performance is usually implemented prior to exposure to dual-task/task switching blocks, it is possible that other factors (apart from block type) contribute to corresponding performance differences. Note that this design decision should likely result in an overestimation (rather than underestimation) of performance in mixed blocks (due to prior training of the individual tasks in constant blocks), while opposite effects, such as those related to fatigue, are less likely to occur given the low number of trials overall. Nevertheless, an ideal design would involve a repetition of single task blocks at the end of the experiment, but at the expense of a reduced number of trials per condition or increased testing time (likely increasing the risk for kinetosis). Future research could address these issues using a more basic simulation environment (e.g., a static driving simulator) which trades ecological validity against the possibility of increased testing duration.

## Summary and conclusions

Taken together, our results indicate that task demands strongly affected cross-modal response control (e.g., regarding the timing and spatial characteristics of eye movements relative to the manual steering responses). Interestingly, the data do not suggest that changes in task demands (addressing the spatial compatibility between oculomotor and manual responses) had strong effects on the manual steering response latency. Specifically, the occurrence of saccades that were spatially incompatible with the required manual responses (i.e., in avoidance instead of approach conditions) did not substantially slow down manual response control, irrespective of block type (constant/mixed). This finding differs from observations made in basic research on cross-modal action control (e.g., Huestegge & Koch, 2009), where cross-modal spatial response incompatibility particularly slowed down manual response latencies. It is possible that the behavioral goal in avoidance conditions is compatible in both effector systems, since steering away from the stimulus is spatially compatible with the intention to eventually execute eye movements that help to guide this intended steering response (see Huestegge & Kreutzfeldt, 2012, and Pfeuffer et al., 2016, for evidence of goal-based oculomotor control). This goal-related compatibility might counteract any spatial incompatibility on more basic motor-related levels. Additionally, it is also possible that both avoidance and approach responses in traffic represent highly trained cross-modal motor routines, and that this training substantially attenuates any spatial motor compatibility effects. In any case, from a practical viewpoint, the lack of such effects appears to be good news indicating that drivers do not necessarily suffer from basic cross-modal response incompatibility issues when avoiding obstacles. Additionally, our data show that mixing task demands had a substantial effect on (initial) error rates and increased the manual (but not oculomotor) RT. We assume that more time is spent on processing the visual information before selecting and executing the steering response in blocks involving switching task demands. Furthermore, the increase of error rates implies that drivers might have difficulties selecting the correct response option within a limited time frame due to conflicting S–R translation rules in approach and avoidance tasks.

In sum, this study provides a first step toward understanding the interaction of oculomotor and manual steering responses in an immersive driving setup by combining methodologies from basic and applied research. Thereby, we demonstrate that while some well-established effects from basic (reduced) research paradigms are transferable to complex realistic settings, others are strongly attenuated or even entirely absent. Overall, this indicates that it is vitally important to take the specific task context, goals, and environmental factors into account when specifying underlying mechanisms of eye–hand interaction.

## Data Availability

On reasonable request via email to the corresponding author, aggregated data used for the analysis presented in this manuscript or raw data can be provided in *csv or *.sav file format.

## Footnotes

1 It is still unknown why and how drivers selected a specific gaze behavior. Specifically, the selection could either be *random* (drivers select randomly between the two different options) or *specific* (because a driver favors a specific option). If the selection is random, no interindividual differences should be observed and all drivers should show both gaze strategies (goal congruent and goal incongruent). However, if the selection is specific, interindividual differences should be observed, and drivers should show a clear preference for one of the two gaze strategies. Corresponding assumptions could also apply with respect to the sequence of the manual and the oculomotor response. Therefore, we conducted a descriptive post-hoc analysis of the gaze strategy and the sequence of the manual and visual response for each driver. This post-hoc analysis indicated no stable interindividual differences of gaze strategy (goal congruent vs goal incongruent) under the instruction “avoid”. However, we found stable interindividual differences for the coordination of the visual and the manual response. In total, seven participants showed an “oculomotor first sequence” more often under both task conditions (constant task vs mixed task). The remaining 13 participants either showed a reversed coordination pattern or no consistent pattern at all. We assume that this difference in the coordination of oculomotor and manual responses and overall task performance could be related to interindividual differences such as personality traits (i.e., Gillath, Canterberry, & Atchley, 2017). However, further research is needed to strengthen this assumption and to identify possible causal factors’ for example, differences in driving experience or personality traits and characteristics.
